# Editorial: Mental health, epidemiology and machine learning

**DOI:** 10.3389/fpsyt.2024.1536129

**Published:** 2025-01-09

**Authors:** Marcos DelPozo-Banos, Robert Stewart, Ann John

**Affiliations:** ^1^ Swansea University Medical School, Swansea, United Kingdom; ^2^ Institute of Psychiatry, Psychology and Neuroscience, King’s College London, London, United Kingdom; ^3^ South London and Maudsley NHS Foundation Trust, London, United Kingdom

**Keywords:** mental health, epidemiology, machine learning (ML), artificial intelligence (AI), research methods, challenges and opportunities

Globally, one in eight people live with a mental health condition, contributing to approximately 16% of disability-adjusted life years (1, 2). The significant impact of mental disorders on quality of life and life expectancy is well established and highlights significant health inequalities.^2^ However, despite this, progress in mental health has lagged behind other medical fields, hindered by social stigma, cultural barriers, resource constraints, and the intrinsic complexity of mental health conditions (2).

Accessing data for mental health research is inherently challenging, due to the relevance of social and environmental factors beyond traditional health systems. Advances in data collection and linkage—including the integration of electronic health records with data from education, employment, and criminal justice—has enabled more comprehensive studies on these determinants (3, 4). However, this new data landscape presents unique analytical challenges. The DATAMIND initiative (https://datamind.org.uk/) aims to optimise the use of UK’s rich mental health data, coordinating research efforts and fostering multidisciplinary collaboration.

Machine learning (ML) has emerged as a promising tool to address these new challenges, offering the power to work with large-scale data resources and produce new insights. However, ML applications in mental health must be rooted in sound epidemiological practices to ensure clinical relevance and to gain the trust of both healthcare community and the public. Our opinion piece (DelPozo-Banos et al.) discussed some of these challenges, particularly: (i) the risk of losing sight of mental health objectives in favour of technical performance; (ii) underlying biases and heightened privacy requirements; and (iii) the difficulties of building, validating and approving ML-enabled clinical devices for mental health disorders with insufficiently clear underlying mechanisms. These ideas, and the setting up of the DATAMIND hub provided impetus for the current Research Topic, titled “*Mental Health, Epidemiology, and Machine Learning*.” With it, we aimed to highlight ML’s potential role in mental health research and to illustrate clinically and epidemiologically sound ML applications in mental health, making the most of novel data sources and linkages.

One of the most evident applications of ML in mental health is in diagnosing complex conditions, enhancing early detection and decision support. Wright-Berryman et al. developed NLP models to identify depression, anxiety, and suicide risk in clinical records; these understandably performed better in cases where symptoms were severe or well-documented. Oh et al. also proposed NLP for depression diagnoses, but their model analyzed the emotional content in patient-psychiatrist interviews. They found that the expression of “disgust” prominently helped to distinguish patients with depression, highlighting the utility of linguistic analysis for capturing emotional markers in mental health diagnostics. Chen et al. presented a decision support tool for ADHD diagnosis, integrating ML with clinical knowledge and processing not only related symptoms, but also comorbid conditions. Their approach pointed to specific features in the Diagnostic Interview for ADHD in Adults that help distinguish ADHD from other conditions, and crucially, their model also identified and flagged complex ADHD cases for expert review. Finally, Merhbene et al. conducted a systematic review on ML for eating disorder detection, revealing challenges such as insufficient data quantity and quality, alongside a lack of representation of minority groups, reduced clinical involvement in development, and culturally driven heterogeneities. Overall, the number and heterogeneity of symptom presentations makes clinical diagnoses a highly complex task in mental healthcare (5), and these papers highlight how ML might be of value to professionals in this regard.

ML can also help to personalise mental health services and treatments to better meet patients’ individual needs. Bernard et al. applied ML clustering to identify usage patterns among young users of a digital mental health platform, with a battery of sensitivity analyses across clustering methods. Their results, validated through hypothesis testing, indicated that user engagement profiles change over time, highlighting the importance of adaptive digital services tailored to changing user behaviors. Garriga et al. developed an ML model that tailors monitoring duration for psychiatric patients with a depression crisis. For over 20% of patients, their model prescribed monitoring beyond the standard one-week period, suggesting that a “one-size-fits-all” approach may overlook important individual needs. Additionally, Yao et al. analysed the satisfaction levels of Chinese psychotherapy patients, identifying cultural factors as critical determinants. While the use of ML for personalised psychiatry is not new (6), it is still under-explored. For example, in their systematic review, Rollmann et al. found only four papers investigating ML applications in psychodynamic psychotherapy, but these foundational models suggest that ML could support tailored treatments, predict treatment responses, and match therapists to patients more effectively. The need for additional research is clear, especially as personalised approaches are critical to improving therapeutic outcomes.

Suicide risk assessment and crisis prediction are areas where ML-driven personalized psychiatry can make a difference in both clinical practice and research. Chou et al. evaluated multiple ML models in a suicide risk identification task based on data from a Japanese population. They found trauma-related emotional distress and functional impairment to be important factors, demonstrating the importance of culturally contextualized risk profiles. Dutta et al. and Wright-Berryman et al. assessed suicide risk using NLP, the former on routinely collected electronic patient records from a mental health service, and the latter on 5-to-10-minute semi-structured interview data. Overall, although ML models may enhance our risk assessment capabilities, they should only be used as complements and not replacements for comprehensive clinical evaluations of patient needs.

Finally, ML can also drive the discovery of new insights on the social and environmental influences on mental health, helping to inform policies and practices. Mason et al. first used NLP to extract indicators of violence from routinely collected clinical notes of a mental healthcare provider. They fed these indicators to an ANN to identify actual experiences of violence. They found that violence-related records were more common among women, mid-life adults, ethnic minorities, and those with PTSD or schizophrenia, highlighting the intersection between demographic and clinical factors. Qasrawi et al. showed that children in violent environments exhibit cognitive and mental health patterns that align with general findings on trauma’s developmental impacts. Castillo-Toledo et al. used NLP to study public perceptions of cocaine use on a large sample of social media posts, providing insights into the way some healthcare professionals openly discussed cocaine’s perceived benefits. These studies demonstrate ML’s capacity to identify and analyze social factors critical to mental health, contributing insights that can shape public health strategies.

In summary, the studies in this Research Topic demonstrate manifold ways in which ML might be of benefit to the field of psychiatry. They maintained a clinical focus and helpfully went beyond simple reporting and comparison of ML performance metrics. They studied the behaviour of such algorithms across varied sub-populations (e.g., by disorder severity) and tried to extract novel clinical insights, aided by additional classical statistical methods. They also openly acknowledged and discussed the limitations of their ML models and sought to validate their findings through traditional epidemiological methods.

Putting all of the above into perspective is Speechley and McTernan’s central work, an opinion piece authored by people with mental health lived experience. In it, they reflected on how ML might help make sense of their lives. They highlighted the need for researchers to foster public trust, cautioning against language that could exacerbate health inequalities and stigma, and emphasizing the need to inform the public that “[their] data saves lives” and how.

Our hope is that this Research Topic serves as a catalyst for deeper conversations on ML’s appropriate role in mental health research and clinical care. Most importantly, researchers must ensure that ML’s transformative potential remains a positive force, advancing mental health research and clinical practice in ways that are ethical, inclusive, and grounded in real-world needs.
